# The network of plants volatile organic compounds

**DOI:** 10.1038/s41598-017-10975-x

**Published:** 2017-09-08

**Authors:** Gianna Vivaldo, Elisa Masi, Cosimo Taiti, Guido Caldarelli, Stefano Mancuso

**Affiliations:** 1 National Research Council, Geosciences and Earth Resources (IGG), Pisa, Italy; 2 Università di Firenze, Dipartimento di Scienze delle Produzioni Agroalimentari e dell’Ambiente (DISPAA), Viale delle Idee, 30, 50019 Sesto Fiorentino (Firenze), Italy; 3 IMT School for Advanced Studies, Piazza San Francesco 19, 55100 Lucca, Italy; 4 Istituto dei Sistemi Complessi (ISC), Roma, Italy; 5 London Institute for Mathematical Sciences, 35a South St. Mayfair, W1K 2XF London, UK

## Abstract

Plants emission of Volatile Organic Compounds (VOCs) is involved in a wide class of ecological functions, as VOCs play a crucial role in plants interactions with biotic and abiotic factors. Accordingly, they vary widely across species and underpin differences in ecological strategy. In this paper, VOCs spontaneously emitted by 109 plant species (belonging to 56 different families) have been qualitatively and quantitatively analysed in order to provide an alternative classification of plants species. In particular, by using bipartite networks methodology from Complex Network Theory, and through the application of community detection algorithms, we show that is possible to classify species according to chemical classes such as terpenes and sulfur compounds. Such complex network analysis allows to uncover hidden plants relationships related to their evolutionary and adaptation to the environment story.

## Introduction

Plants produce an amazing variety of metabolites but only a few of these are involved in “primary” metabolic pathways, that is to say common to all organisms. Others (indicated as “secondary” metabolites) are instead characteristic of smaller plants groups^1^. Indeed, such “secondary” metabolites^2^ are the result of different plants responses, through the course of evolution, to specific needs. Among such metabolites, volatile organic compounds (VOCs) play a dominant role^3^. Being released by almost any kind of tissues^4, 5^ and type of vegetation (trees, shrubs, grass, etc.) as green leaf volatiles, nitrogen-containing compounds and aromatic compounds, plants VOCs can be emitted constitutively^6, 7^ or in response to a variety of stimuli. They are involved in a wide class of ecological functions, as a consequence of the interactions of plants with biotic and abiotic factors^8^. Plants use VOCs to perform a variety of tasks, as different as: indirect plant defence against insects^9^; pollinators attraction^10^; plant-to-plant communication^11, 12^; thermo-tolerance and environmental stress adaptation^7^; defence from predators^13^.

According to their biosynthetic origin and chemical structure, plant volatiles can be grouped into isoprenoids or terpenoids, but also oxygenated VOCs (OVOCs), such as methanol (CH_4_O), acetone (C_3_H_6_O), acetaldehyde (C_2_H_4_O), methyl-ethyl-ketone (MEK, C_4_H_8_O) and methyl-vinyl-ketone (MVK, C_4_H_6_O)^14^; in few cases, sulfur compounds (e.g. in Brassicales) and furanocoumarins and their derivatives (e.g in Apiales, Asterales, Fabales, Rosales) are also found^15, 16^.

Interestingly, VOCs emissions strongly depend on the species (see ref. 17 for references). Indeed, different plant lineages often adopt different chemical solutions to face the same problem; this is the case, for example, of the different odorous volatiles emitted by different flowers for solving the common problem of attracting the same type of pollinator, which usually visit a large amount of plant species^2^.

In this paper we apply complex networks analyses^18–20^ and in particular community detection procedures^21, 22^ to identify community structures in plants species network, on the basis of their similarities in terms of VOCs emissions. Complex Network theory^23–25^ has been already successfully used in ecology to determine, for example, the stability and robustness of food webs^26^ with respect to the removal of one or more individuals from the network, or in biology to study the structure of protein interactions in the cell by the so-called protein interaction networks (PINs)^27^, similarly metabolic networks are used to study the biochemical reactions which take place into living cells^28^. Furthermore, biological networks found important applications in medicine^29^, where they are applied as a solution to human diseases comorbidity analyses^30^, or to study the structural and functional aspects of human brain, by defining the reciprocal interactions of the cerebral areas^31^. With respect to the above activities, the application of Complex Networks Theory in botany is still scarce, apart some tentatives of comparing different ecosystems looking for steady (i.e. “universal”) behaviours^32^. Recent applications of graph theory in botany deal with the attempt of assessing plants species similarities on the basis of both their diaspore morphological properties, and fruit-typology ecological traits^33^. Following the same approach, in this paper we perform network analyses with the goal of identifying communities of “similar” species, starting from their VOCs, a proxy of the different ways in which different species react to external stimuli.

To this purpose, we arrange data in bipartite graphs, a method particularly suitable to study the relations between two different classes of objects and to group individuals according to the properties they share. In a bipartite graph vertices can be divided into two disjoint sets, such that every vertex of one set is connected only with a vertex of the other set. In this way no links are present between vertices of the same set. In our case, the plants species and their VOCs define the two independent sets of vertices built from botanical data. Bipartite graphs are then analysed by considering the two different projections of vertices of the same class. In this way we have a first graph by considering all plants species (as vertices) connected on the basis of the emitted VOCs and a second graph made up by VOCs connected if the two are produced by the same plants. On thee two graphs we perform a community detection to create a taxonomic tree^22^.

## Results and Discussion

The present research work focuses on a group of 75 VOCs emitted by 109 different plant species in basal conditions, in order to understand if taxonomy-related plants emit a similar VOC composition. To assure the analysis to be robust and consistent, we measured the VOCs emitted by each plant species by a three times replication experiment (we refer to the section “Materials and methods” for a detailed description of the dataset preparation). Complex networks analysis is then applied to the VOCs dataset represented as bipartite network, in order to easily define metrics and hidden statistical properties able to discriminate and classify plant taxonomy based on VOCs patterns.

### Data preprocessing

The 109 plants species analysed are representative of 56 families, and the dataset is quite homogeneous in terms of families percentages. The most copious families are: *Asteraceae* (8.26%), *Solanaceae* (6.42%), *Rosaceae* (6.42%), *Fabaceae* (5.5%), *Brassicaceae* (4.59%), and *Polygonaceae* (3.67%). All the other families are present at lower percentages.

**Figure 1:**
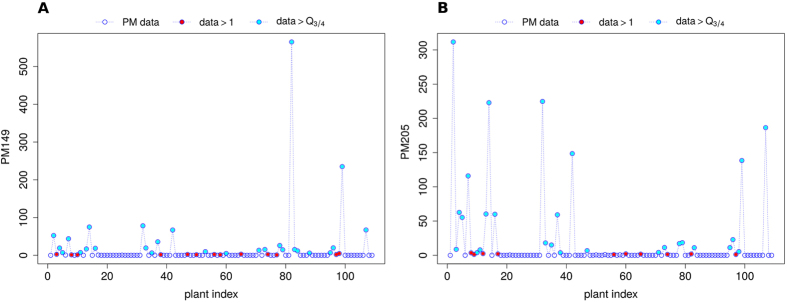
PM149 (Tp/STp-f) (panel (A)) and PM205 (STp) (panel (B)) emissions. Protonated mass data are represented by empty blue bullets. Red dots correspond to values larger than 1, while cyan dots refer to those data exceeding ${Q}_{\tfrac{3}{4}}$. x-axis: plants index. Protonated masses are expressed as mass-to-charge (m/z) ratios.

To evaluate the data statistical structure we plotted for each protonated mass the emission recorded for all the 109 plants species. Figure 1 (empty blue bullets) shows the emission of protonated masses PM149 (panel A) and PM205 (panel B), as two examples of VOCs records behaviour. Protonated masses are expressed as mass-to-charge (m/z) ratios. From the chemical composition point of view, PM149 and PM205 belong to terpenes/sesquiterpenes fragments (Tp/STp-f) and sesquiterpenes (STp) classes, respectively. The VOCs series turn out to be characterised by the superposition of an irregular, abruptly changing pulsatile component and a slowly changing one. More in details, zero-values indicate the lack of emission of that specific VOC for the corresponding plants, and the flat and uniform plateau suggests a small emission of the same VOC. Finally, spike-like pulses, clearly emerging from the background, are related to a huge emission of that VOC for a given plant. Figure 1 suggests that both the protonated masses PM149 and PM205 are emitted in large quantity just by few species. That behaviour turned out to be representative of the whole dataset (not shown).

**Figure 2:**
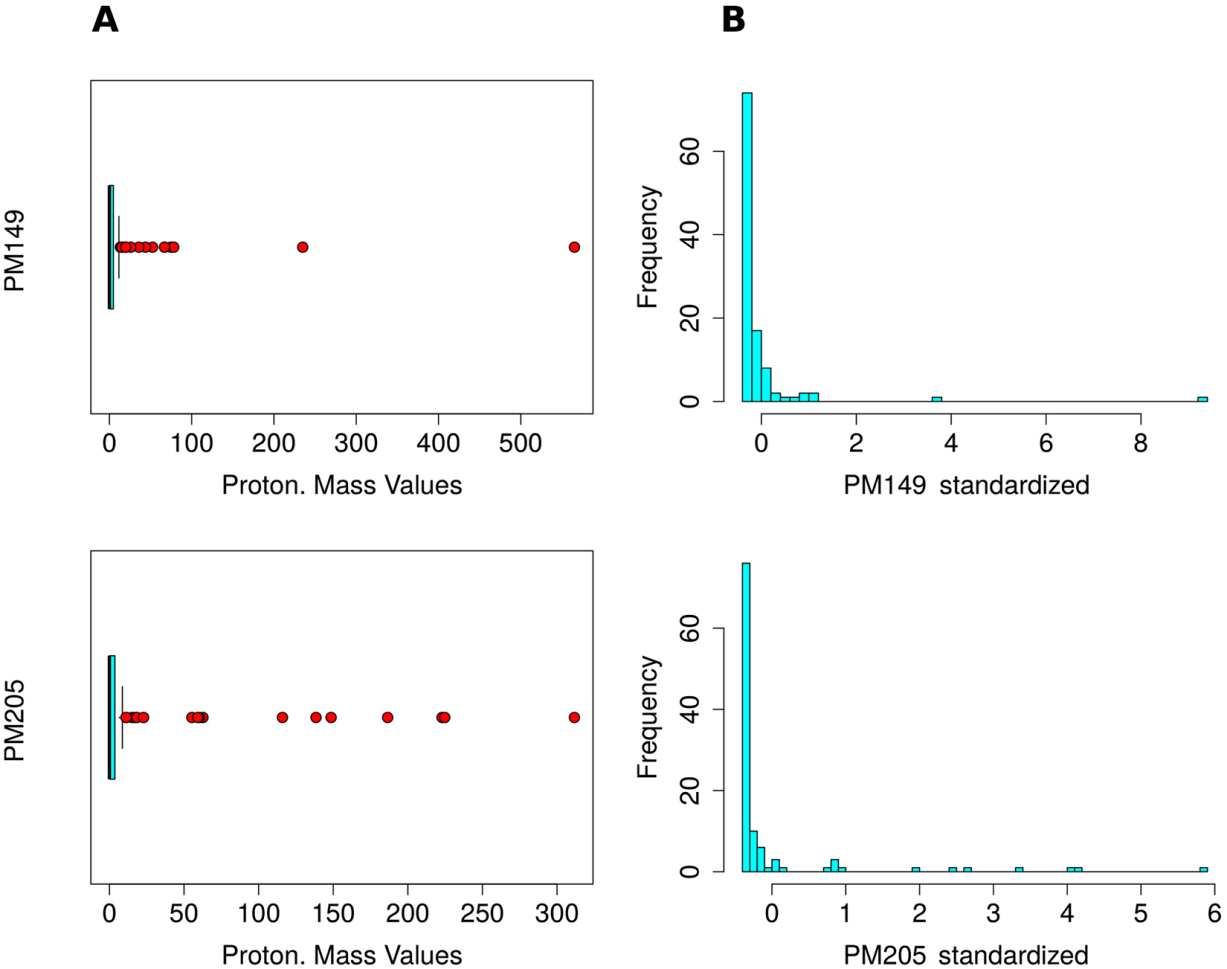
PM149 (Tp/STp-f) and PM205 (STp) emissions. Panels (A) boxplots, *IQR* (cyan rectangles) and outliers (red dots). Panels (B) Absolute frequency histograms (y-axes) versus data standardised values (x-axes), obtained by removing the sample mean, and by normalizing the residuals to the sample standard deviation. Protonated masses are expressed as mass-to-charge (m/z) ratios.

From a statistical point of view the same result was confirmed by the presence of outliers inside each record, that can be easily visualised by boxplot methodology^34, 35^ (see Section “Materials and methods” for more details). Outliers are shown in Fig. 2 (panels A, red dots), and they correspond to those observations far from the sample mean. In that case, since the behaviour was coherent for all the VOCs, we excluded the presence of outliers as a consequence of merely experimental errors. Rather, protonated mass records were characterised by heavy-tailed distribution, as Fig. 2 (panels B) shows: few values lie in the queues of the absolute frequencies sample data histograms. In that figure, standardised values have been employed in order to assure the results comparability among PM149 and PM205.

Notwithstanding the clear dominating behaviour of some species emissions with respect to the other plants, for a given VOC (i.e. the outliers described in the previous paragraph), the statistical procedure that takes into account just the highest recorded values among the outliers (i.e., the extreme values) turned out to be too restrictive. Actually, not even a small emission of a protonated mass can be neglected from an experimental point of view. A low emission can also be a signal from a wounded leaf, and it has to be evaluated when comparing the species reciprocal behaviour with respect to an external wounding perturbation.

### Basic network analysis

In order to investigate the relationships among plants according to their VOCs emissions, we consider bipartite graphs properties which turn out to be particularly suitable to solve communities detection issues. The final goal of our analysis is to cluster plants in a relatively small number of statistically stable communities, each one made up by those plants emitting the same VOCs and to see if this reveals hidden properties of the species analysed. Standard clustering methodologies have been taken into account as a quantitative guide for a more critical analysis. In the binary analysis all species have been considered irrespective of the strength of their emissions. At the same time, all VOCs were equally taken into account during the analysis of the emerging plants network structure. A detailed description of the applied methodology can be found in the section “Materials and methods”.

To represent in a suitable way the connections and relationships between the available plants species starting from our experimental data, we considered two different ways of building the plants network, depending on the statistical measure used to represent the highly not-Gaussian behaviour of the series. In the first case, we set a fixed threshold for the signal intensity (1 normalised counts per second, *n*cps) and we considered significant all the emissions larger than it (graph: *G*_1_(*V*, *E*)). In the second case, we applied a more severe criterion, and we decided to take into account just the emissions above the third quartile ${Q}_{\tfrac{3}{4}}$ of the corresponding data statistical distribution (graph: *G*_2_(*V*, *E*)). Figure 1 shows both the approaches applied to PM149.1 (panel A) and PM205.1 (panel B). Red dots in both panels highlight values larger than 1, while cyan bullets represent the value exceeding ${Q}_{\tfrac{3}{4}}$.

According to the case we create two distinct bipartite graphs (*G*_1_(*V*, *E*) and *G*_2_(*V*, *E*), respectively as indicated above), made up by *V* = 184 vertices and divided into two layers (or set of vertices): the first category of vertices is made up by the *V*_*P*_ = 109 plants species and the second one is composed by the *V*_*PM*_ = 75 emitted VOCs. Intuitively, as the name suggests, a bipartite graph is composed by qualitatively two different sets of vertices. The first layer is made up by the individuals/entities (*V*_*P*_ in our case, the plants), and the second one is composed by the properties they share (*V*_*PM*_, in our case the VOCs). In this way, by connecting each vertex of the first set to all its properties in the second set, we can create a 2-layer-network. Plants-to-plants species networks are subsequently defined by considering as vertices the plant species in the database. From a mathematical point of view, that approach translates into computing the bipartite projection of both *G*_1_(*V*, *E*) and *G*_2_(*V*, *E*). Two vertices are connected if they share at least one common property, in other words, two plants are connected in the network, if they emit almost the same amount of a specific VOC. For every network, we considered size (number of edges), order (number of vertices), degree (average and its distribution), density (the ratio of actual vertices against the possible ones), clustering and finally the community structure.

#### Threshold-based graph

The plants graph corresponding to the first method was created as a bipartite projection of graph *G*_1_(*V*, *E*). In the resulting graph ${G}_{1}^{P}({V}_{1},\,{E}_{1})$ plants are interconnected on the basis of the common VOCs they emit, and an emission is relevant if it overcomes the established threshold of 1 *n*cps. ${G}_{1}^{P}({V}_{1},\,{E}_{1})$ is made up of *V*_1_ = *V*_*P*_ = 109 vertices (plant species), and *E*_1_ = 5,886 edges. Species *i* and species *j* are linked if they share at least one common emitted protonated mass. The weight *w*_*ij*_ of each link *e*_*ij*_ is given by the total number of shared VOCs between species *i* and species *j*. ${G}_{1}^{P}({V}_{1},\,{E}_{1})$ is a fully connected graph, its density $D=\frac{2{E}_{1}}{{V}_{1}({V}_{1}-\mathrm{1)}}$ is equal to 1, and the degree of each node is equal to 108, which is also equal to the nodes mean degree $(\overline{k}=\tfrac{1}{{V}_{1}}\,{\sum }_{i=1}^{{V}_{1}}\,{k}_{i}=\tfrac{2{E}_{1}}{{V}_{1}})$. It follows, from the network analysis, that each vertex is connected to all the other vertices, or equivalently each species emits at least one VOC in common with all the other species.

That network structure is poorly able to extract information about the dominant behaviour of one species with respect to the others, in terms of their emissions. Concerning the links weights distribution, the maximum number of protonated masses shared by two species is 66, and in average species are connected by links of weight *w*_*ij*_ = 24, in agreement with the dense structure of the network.

#### Third-quartile-based graph

The plants graph corresponding to the second test was analogously constructed as the species-vs-species bipartite projection graph of *G*_2_(*V*, *E*) graph. Again, the common emitted VOCs determine the presence or not of a (weighted) link between two nodes. ${G}_{2}^{P}({V}_{2},\,{E}_{2})$ is made up by *V*_2_ = *V*_*P*_ = 109 vertices (plants species) and *E*_2_ = 2,343 edges. Links are less by construction: in that case, for each VOC just the emissions larger than ${Q}_{\tfrac{3}{4}}$ were considered significant. It follows that the network construction procedure accounted for a more severe pruning. Graph density reduces to 0.39, turning out to be consistent with the fact that the graph is not fully connected. Rather, isolated vertices emerge, suggesting the presence of plants which do not emit any of the measured VOCs at a high level. By removing them the graph density increase to 0.73.

**Figure 3:**
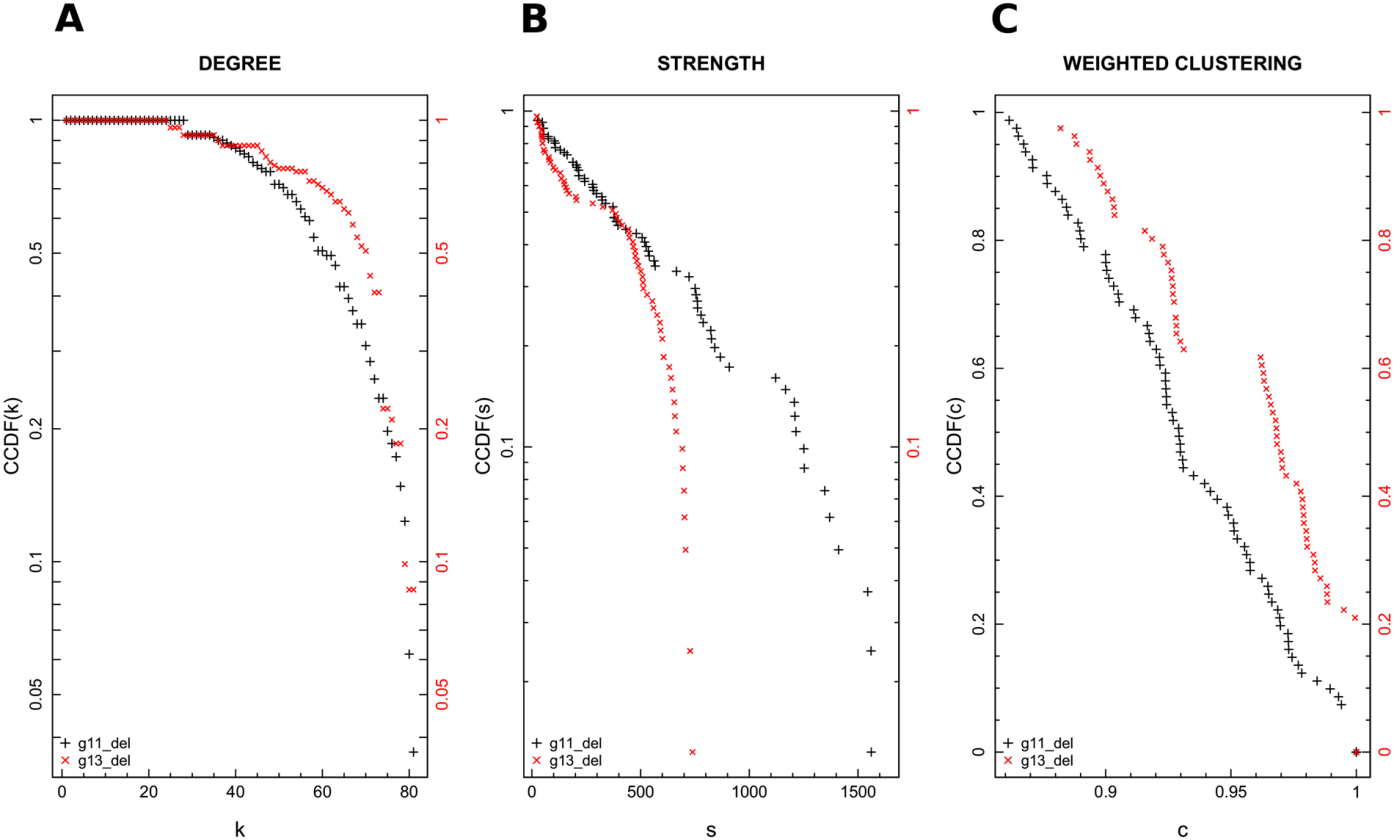
Complementary cumulative distribution functions (CCDF) of degree and strength are reported in log-line scale in panel (A,B), respectively, for both ${G}_{2}^{P}({V}_{2},\,{E}_{2})$ (black crosses) and ${G}_{3}^{P}({V}_{3},\,{E}_{3})$ (red crosses). Panel (C) moreover, shows weighted clustering coefficient distribution. More precisely, CCDF (on y-axis) is plotted versus the weighted clustering parameter (x-axis) on linear scale. The isolated nodes were not taken into account for the corresponding network’s basic metric analysis.

In that case the majority of species share few common VOCs emitted (i.e. the mean of the edges weights is around 5). On the contrary, some vertices are connected by heavy links (the maximum weight’s value is 67, similarly to the previous case). Figure 3 shows some of the standard networks metrics, which are described into details in paragraph “Basic network analysis” of section “Materials and methods”. Figure 3 (panel A, black crosses) shows the network degree distribution *P*(*k*), representing the fraction of vertices with degree *K* > *k*. A log-line plot is chosen to display the degree complementary cumulative distribution function (CCDF). The graph strength distribution is also shown in Fig. 3 (panel B, black crosses) in log-line scale. The strength *s* of a vertex corresponds practically to its weighted degree, thus it takes into account the total weight of the vertex connections, and it allows one to identify high and low concentration edges-regions inside an undirected graph. The maximum strength value is equal to *s*_*max*_ = 1,624 and it corresponds to *Lavandula spica* L. (Lavender) species, while the minimum value is equal to *s*_*min*_ = 27 and it is common to *Humulus lupulus* L. (Wild hop), *Actinidia arguta* (Siebold & Zucc.) (Hardy kiwi), *Ficus benjamina* L. (Weeping fig), *Magnolia liliiflora* (Desr.) (Japanese magnolia), and *Diospyros lotus* L. (Date-plum) species. Finally, Fig. 3 (panel C) shows the local clustering coefficient, defined as the tendency among two vertices to be connected if they share a mutual neighbour.

Taken as a whole, Fig. 3 suggests that plants network is not dominated by some central nodes (or hubs) characterised by a huge amount of connections linking them to all the other less connected vertices. By resorting to complex networks formalism, the plants network is not a scale-free one since its degree distribution is not highly heterogeneous. Notwithstanding, some species emit a large quantity of VOCs and communities detection algorithms are applied to identify them and the respective aggregating VOCs. The graph ${G}_{2}^{P}({V}_{2},\,{E}_{2})$ isolated nodes were removed before performing that basic metrics analysis for visual reasons. The degree and strength of an isolated node are equal to 0 by definition and the clustering coefficient is not defined.

#### Selected-VOCs graph

A third test was performed on a reduced version of the original database. All those volatiles strictly associated to the mechanical wounding^36, 37^ possibly made during the preparation of the experimental samples were excluded. Indeed, the volatiles produced in consequence of wounding tend to be quite conserved among different species^38, 39^; their inclusion in the database could lead to misinterpretation. Furthermore, other compounds that turned out to be less powerful in the aggregation features, as highlighted by the above described analyses, were removed from the dataset. As a result, a selection of 30 protonated masses was taken into account.

In order to compensate the filter introduced by that selection of relevant VOCs, a threshold equal to 0 *n*cps was used to distinguish between relevant and negligible emissions of a specific VOC: all the emissions larger than 0 *n*cps were considered comparable. The corresponding bipartite network *G*_3_(*V*, *E*) was made up by *V* = 139 vertices subdivided in two sets: *V*_*P*_ = 109, i.e. the plants analogously to previous graphs, and *V*_*PM*_ = 30 (i.e., the selected protonated masses). In order to study plants network, the bipartite projection ${G}_{3}^{P}({V}_{3},\,{E}_{3})$ was analysed. The vertices are still *V*_3_ = *V*_*P*_ = 109, while the edges are equal to *E*_3_ = 2,522, similarly to the third-quartile-based graph ${G}_{2}^{P}({V}_{2},\,{E}_{2})$. The graph density is 0.43 due to the presence of 28 isolated nodes, while it raises to 0.78 if they are removed.

By following the previous approach for estimating the graph basic metrics, Fig. 3 (panel A, red crosses) shows ${G}_{3}^{P}({V}_{3},\,{E}_{3})$ complementary cumulative degree distribution *P*(*k*), while Fig. 3 (panel C, red crosses) depicts the graph strength distribution. Both figures are in log-line scale. The network strength maximum value decreases to *s*_*max*_ = 746, but it still corresponds to *Lavandula spica* L. (Lavender) species, which again emerges as the most connected node. On the other side, the strength minimum value is *s*_*min*_ = 23 for *Cyperus papyrus* L. (Papyrus), *Salicornia europaea* L. (Glasswort), and *Solanum quitoense* Lam. (Naranjilla) species. Further, ${G}_{3}^{P}({V}_{3},\,{E}_{3})$ is characterised by a smaller range of strength values with respect to ${G}_{2}^{P}({V}_{2},\,{E}_{2})$, and a more restricted set of nodes seem to dominate the network behaviour. Nevertheless, the graph degree and strength distribution do not suggest the presence of a scale-free structure behind our data. Finally, Fig. 3 (panel C, red crosses) shows ${G}_{3}^{P}({V}_{3},\,{E}_{3})$ clustering coefficient. The behaviour is similar to the one observed for ${G}_{2}^{P}({V}_{2},\,{E}_{2})$ graph. Such as for ${G}_{2}^{P}({V}_{2},\,{E}_{2})$ graph, isolated nodes were removed before performing that basic metrics analysis. Analogously, the strength minimum value is performed after excluding the isolate nodes, since the degree *k* and thus the strength *s* of an isolated node are equal to 0 by definition.

### Community detection analysis

#### Threshold-based and third-quartile-based graphs

A first attempt to group plants on the basis of the VOCs emitted was performed by applying the community detection to both the dense ${G}_{1}^{P}({V}_{1},\,{E}_{1})$ graph and the third-quartile-based graph ${G}_{2}^{P}({V}_{2},\,{E}_{2})$. For both of them, subgraphs were obtained filtering-out a growing number of links, from the lower to the higher weighted ones. A unit-based normalization was applied to edges weights to limit their values to the [0, 1] range (${w}_{ij}^{resc}$ parameter in Table 1). Four communities detection algorithms were applied: (i) Louvain or Blondel’s modularity optimization algorithm (BL), (ii) fast greedy hierarchical agglomeration algorithm (FG), (iii) walktrap community finding algorithm (WT), and (vi) label propagation community detection method (LP). We refer to the section “Materials and methods” for a detailed description of the communities detection methods.

**Table 1:** Communities detection of graph ${G}_{1}^{P}({V}_{1},\,{E}_{1})$ by fast greedy (FG), walktrap algorithms (WT), Blondel modularity optimization (BL), and label propagation (LB).

FG	WT	BL	LP	${{\boldsymbol{w}}}_{{\boldsymbol{ij}}}^{{\boldsymbol{resc}}}$	E	N	is.connected	density
2	2	2	1	0	5886	109	TRUE	1
2	2	2	1	0.1	5303	104	TRUE	0.99
2	2	2	1	0.2	4776	101	TRUE	0.95
2	2	2	1	0.3	3220	85	TRUE	0.9
2	2	2	1	0.4	1316	58	TRUE	0.8
2	1	3	1	0.5	697	44	TRUE	0.74
2	28	2	1	0.6	309	28	TRUE	0.81
2	4	2	1	0.7	156	21	TRUE	0.74
2	12	2	1	0.8	48	12	TRUE	0.73
2	3	2	1	0.9	13	7	TRUE	0.62
1	2	1	1	1	1	2	TRUE	1

Several filtered-by-edges-weight versions of the graph were analysed (one for each row). Graph edges weight values are normalised to the interval $[0,1]$.

Notwithstanding some discrepancies in the results depending on algorithms optimization after pruning the network, two big communities emerge from ${G}_{1}^{P}({V}_{1},\,{E}_{1})$ analysis, which turned out to be robust to algorithm changes and to the filtering procedure of the edges weights (see Table 1), exception made for severe filters (rescaled weight parameter *w*_*ij*_ > 0.5 in Table 1). In that case, almost half of the graph nodes were filtered out, thus reducing the reliability of the related results as the consequence of a huge loss of information. On the contrary, by pruning the graph from the most heavy links, the results were statistically comparable, thus meaning that the plants network was not dominated by some big vertices acting as hubs of the whole system. The two uncover communities embed the 61.47% and 38.53% of the total amount of species inside the database, respectively.

**Figure 4:**
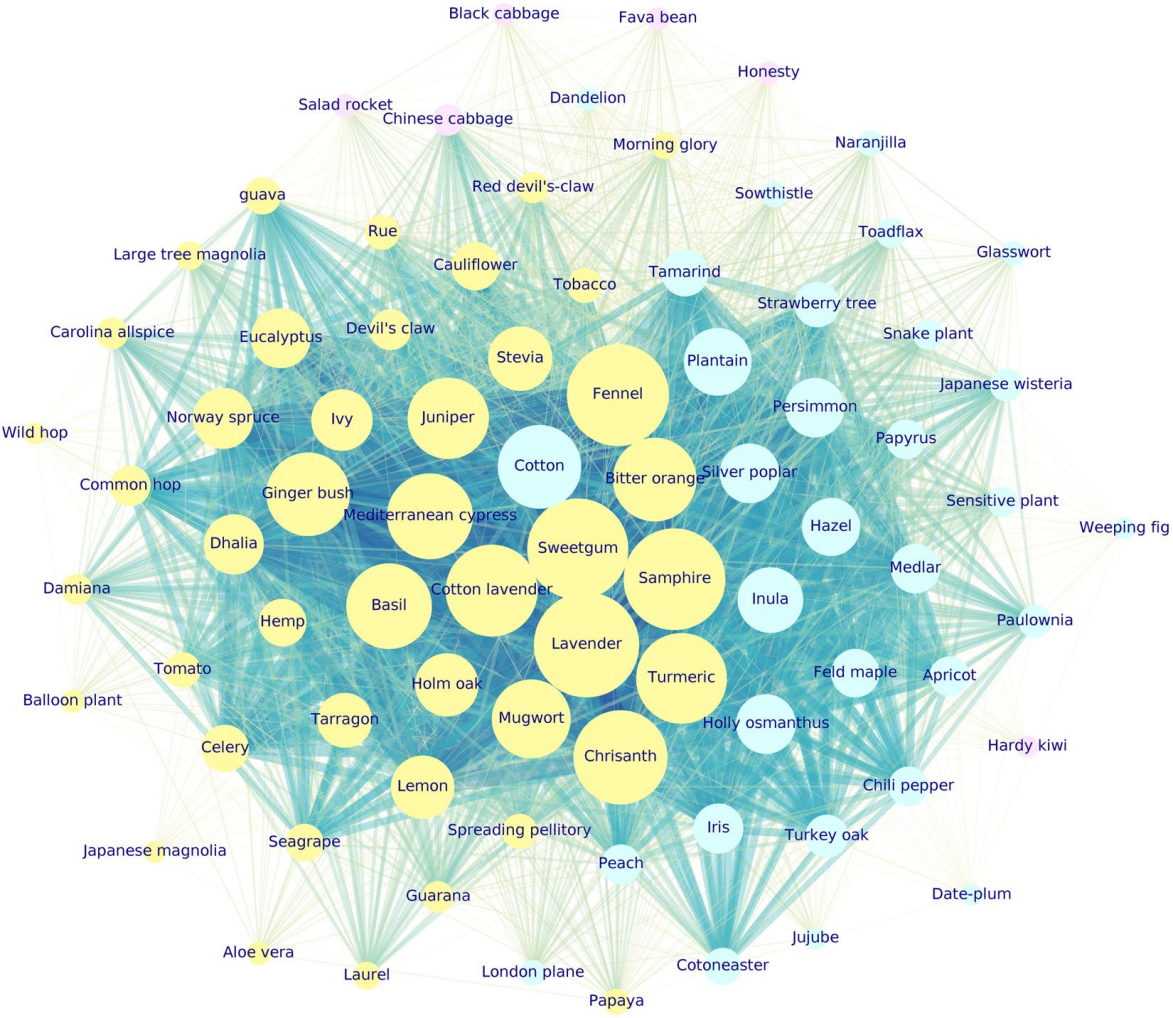
${G}_{2}^{P}({V}_{2},\,{E}_{2})$ third-quartile-based graph. Each color corresponds to one detected community: cluster 1 (yellow), cluster 2 (aqua), cluster 3 (violet). The 28 isolated nodes are not shown (cluster 4). Nodes dimension is proportional to nodes weighted degree. Edges thickness is proportional to the edges weight. *Lavandula spica* L. (Lavander), *Foeniculum vulgare* Mill. (Fennel), *Crithmum maritimum* L. (Samphire), *Liquidambar styraciflua* L. (Sweetgum), visible as biggest yellow nodes, are some of the most active species in terms of VOCs emissions.

The situation improved by analyzing the communities of ${G}_{2}^{P}({V}_{2},\,{E}_{2})$ graph. Figure 4 is a representation of ${G}_{2}^{P}({V}_{2},\,{E}_{2})$ plants network. The dimension of each node is proportional to the node’s weighted degree. The thickness of each link connecting two nodes *i* and *j* is proportional to the link’s weight, *w*_*ij*_. Nodes colours refer to cluster membership.

In that case two big and one small clusters emerge from a basic community detection. The two big clusters embed 44 and 31 species, i.e. respectively the 40.4% and 28.4% of the species present in the dataset (yellow and aqua clusters in Fig. 4). The third small cluster (6 species accounting for the 5.5% of the species dataset, violet cluster of Fig. 4) is made up by *Brassicaceae* family mainly, exception made for the *Brassica oleracea* L. var botrytis species (Cauliflower) which belongs to another community (yellow cluster in Fig. 4). By construction 28 isolated nodes emerged (not shown in Fig. 4), corresponding to species which were not sharing any of the measured VOCs with the other plants. Isolated nodes accounted for the 25.7% of species total amount. Again, the results were consequent to the simultaneous application of more than a single methodology. The findings proved to be independent from the applied methodology and they were considered robust and reliable from a statistical point of view. Hereafter, the composition of every cluster is summarised, together with the protonated mass that the species share at graph’s communities level:

- cluster 1: 31 species (28.4% of the database total species) grouped in 21 families; prevailing families: *Rosaceae*, *Asteraceae*, *Fabaceae*, *Ebenaceae*, *Plantaginaceae*, and *Solanaceae*. Two VOCs in particular are responsible for that partitioning: PM27 (hydrocarbons, Hyd) and PM73 (acids, A) (20 species), followed by PM55 (aldehydes fragment, Ald-f), PM89 (esters, E), PM115 (acids, A) (19 species), and PM53 (fragment, f), PM81 (aldehydes fragments, Ald-f) (18 species). In general, the more informative VOCs for this cluster are compounds belonging to several chemical classes. Notice that from *m*/*z* = 123 (PM123) to *m*/*z* = 205 (PM205), where peaks deriving from terpenes, sesquiterpenes and their fragments are found, the emissions are null for all the species. One species can emit more than one VOC, so that all the species can be counted more than once to assess how many species share the same protonated mass emission. *Gossypium herbaceum* L. (Cotton), *Plantago lanceolata* L. (Plantain), and *Inula viscosa* L. (Inula) species are between the highest weighted degree nodes in Fig. 4.
- cluster 2: it is the biggest community, made up of 44 species (40.4% of the total species amount) grouped into 27 families; dominant families: *Asteraceae*, *Apiaceae*, *Cannabaceae*, *Lamiaceae*. The species belonging to that cluster emit, taken as a whole, a large amount of VOCs. They share in particular the emission of VOCs which are or refer to terpenes compounds, which are among the principal odour-like molecules emitted by plants flowers and leaves. In details, 28 species share PM123 and PM135, both terpenes or sesquiterpenes fragments (Tp/STp-f); 27 species share PM93 (Tp-f), PM95 (STp-f), PM105 (heterocyclic aromatic compounds, HeArC), PM109 (Tp-f), PM119 (Tp-f), PM121 (Tp-f), PM137 (Tp/STp-f), PM143 (ketones and aldehydes, K/Ald), PM149 (Tp/STp-f), PM163 (STp-f), PM205 (STp); 26 species share PM91 (hydrocarbons, Hyd), PM107 (HeArC), PM111 (aldehydes, Ald), PM153 (Tp-f).
  Accordingly, that community includes plant species characterised by intense flavour, such as *Lavandula spica* L. (Lavander, a well known plant used for its flavour), *Foeniculum vulgare* Mill. (Fennel, an anise-flavored spice), *Crithmum maritimum* L. (Samphire, a very flavoured sea fennel), and *Liquidambar styraciflua* L. (Sweetgum, commonly used as flavor and fragrance agent). A more detailed description of cluster 2 is supplied hereafter.
- cluster 3: 6 species only (5.5% of total species) from 3 families: *Brassicaceae* (dominating family with 4 species), *Actinidiaceae*, and *Fabaceae*. Interestingly, the *Brassicaceae* Cauliflower belongs to the previous community (i.e., to cluster 2, where species characterised by more intense odours and presence of terpenes compounds are clustered). Indeed, Cauliflower is, among the *Brassicacaeae* species included in the present study, one of the richest in VOCs and terpenes^40, 41^. This is the most homogeneous community in terms of family composition. PM63, a typical sulfur compound (SC), is the most emitted VOC, being released by 5 species (4 of them belonging to the *Brassicaceae* family), followed by another sulfur compound, PM49, and PM83 (alcohols fragment, Alc-f) (3 species), PM87 (Ald/Alc). In particular *Brassica rapa* L. (Chinese cabbage) emits also PM85 (Alc-f), PM103 (esters, E), PM117 (Alc), PM129 (Alc), PM143 (ketones and aldehydes, K/Ald). The latter protonated mass, tentatively identified as 2-Nonanone^42^ has been already reported in Chinese cabbage^43^. The emission of all the other VOCs is null for the whole species set.
- cluster 4: 28 isolated species (25.7% of total species) belonging to 20 different families dominated by *Polygonaceae*, *Rosaceae*, *Solanaceae*, *Araceae*, *Fabaceae*. They do not share any emitted VOC with other plants, since they do not release any protonated mass at all. That result has to be interpreted taking into account ${G}_{2}^{P}({V}_{2},\,{E}_{2})$ construction procedure. Just the emissions exceeding the ${Q}_{\tfrac{3}{4}}$ of the corresponding protonated mass distribution were considered as relevant. In that sense that nodes are isolated from the rest of the graph and they do not emit VOCs.

Previous results are summarised in Table 2, which shows the dominant families in each cluster and how many species belong to that families. The list of species present in each cluster is reported in Table 3.

**Table 2:** Plants families composition in each community extracted from third-quartile-based graph ${G}_{2}^{P}({V}_{2},\,{E}_{2})$ by modularity (BL) algorithm, and the corresponding amount of species belonging to that families for each community.

cluster 1		cluster 2		cluster 3		cluster 4	
Rosaceae	4	Asteraceae	5	Brassicaceae	4	Polygonaceae	3
Asteraceae	3	Apiaceae	3	Actinidiaceae	1	Rosaceae	3
Fabaceae	3	Cannabaceae	3	Fabaceae	1	Solanaceae	3
Ebenaceae	2	Lamiaceae	3			Araceae	2
Plantaginaceae	2	Cupressaceae	2			Fabaceae	2
Solanaceae	2	Magnoliaceae	2			Apocynaceae	1
Amaranthaceae	1	Martyniaceae	2			Aquifoliaceae	1
Asparagaceae	1	Myrtaceae	2			Asteraceae	1
Betulaceae	1	Rutaceae	2			Crassulaceae	1
Cyperaceae	1	Sapindaceae	2			Faboideae	1
Ericaceae	1	Solanaceae	2			Hydrangeaceae	1
Fagaceae	1	Araliaceae	1			Iridaceae	1
Iridoideae	1	Brassicaceae	1			Lauraceae	1
Malvaceae	1	Calycanthaceae	1			Lythraceae	1
Moraceae	1	Caricaceae	1			Malvaceae	1
Oleaceae	1	Composite	1			Moraceae	1
Paulowniaceae	1	Convolvulaceae	1			Oleaceae	1
Platanaceae	1	Fagaceae	1			Poaceae	1
Rhamnaceae	1	Hamamelidaceae	1			Portulacaceae	1
Salicaceae	1	Lauraceae	1			Vitaceae	1
Sapindaceae	1	Pinaceae	1				
		Polygonaceae	1				
		Rutacee	1				
		Turneraceae	1				
		Urticaceae	1				
		Xanthorrhoeaceae	1				
		Zingiberaceae	1				
**Fam**.	# **Spec**.	**Fam**.	# **Spec**.	**Fam**.	# **Spec**.	**Fam**.	# **Spec**.

Exception made for cluster 3 (violet), a huge families heterogeneity characterises all the other communities.

**Table 3:** Species composition of each cluster found in ${G}_{2}^{P}({V}_{2},\,{E}_{2})$ third-quartile-based graph.

cluster 1		cluster 2		cluster 3		cluster 4	
*Mimosa pudica* L.	Fabaceae	*Ocimum basilicum* L.	Lamiaceae	*Brassica rapa* L.	Brassicaceae	*Zamioculcas zamiifolia* (Lodd.)	Araceae
*Cyperus papyrus* L.	Cyperaceae	*Brassica oleracea* L. var botrytis	Brassicaceae	*Brassica oleracea* L. var acephala	Brassicaceae	*Rheum rhabarbarum* L.	Polygonaceae
*Ziziphus jujuba* Mill.	Rhamnaceae	*Stevia rebaudiana*	Asteraceae	*Actinidia arguta* (Siebold & Zucc.)	Actinidiaceae	*Hydrangea macrophylla* (Lam.)	Hydrangeaceae
*Platanus x Acerifolia* (Willd.)	Platanaceae	*Cannabis sativa* L.	Cannabaceae	*Eruca sativa* (Mill.)	Brassicaceae	*Solanum marginatum* L.	Solanaceae
*Plantago lanceolata* L.	Plantaginaceae	*Nicotiana tabacum* L.	Solanaceae	*Vicia faba* L.	Fabaceae	*Persea americana* Mill.	Lauraceae
*Arbutus unedo* L.	Ericaceae	*Eucalyptus globulus* L.	Myrtaceae	*Lunaria annua* L.	Brassicaceae	*Vitis vinifera* L.	Vitaceae
*Cotoneaster horizontalis* Decne.	Rosaceae	*Ibicella lutea* L.	Martyniaceae			*Echeveria elegans* (Rose)	Crassulaceae
*Sonchus oleraceus* L.	Asteraceae	*Proboscidea parviflora* (Woot. & Standl.)	Martyniaceae			*Arundo donax* L.	Poaceae
*Inula viscosa* L.	Asteraceae	*Quercus ilex* L.	Fagaceae			*Rumex acetosella* L.	Polygonaceae
*Corylus avellana* L.	Betulaceae	*Artemisia dracunculus* L.	Composite			*Acacia dealbata* Link	Fabaceae
*Prunus armeniaca* L.	Rosaceae	*Convolvulus cneorum* L.	Convolvulaceae			*Robinia pseudoacacia* L.	Faboideae
*Acer campestre* L.	Sapindaceae	*Juniperus communis* L.	Cupressaceae			*Olea europaea* L.	Oleaceae
*Osmanthus heterophyllus* (G. Don)	Oleaceae	*Santolina chamaecyparissus* L.	Asteraceae			*Fragaria vesca* L.	Rosaceae
*Diospyros kaki* L.	Ebenaceae	*Apium graveolens* L.	Apiaceae			*Rosa chinensis* (Jacq.)	Rosaceae
*Ficus benjamina* L.	Moraceae	*Ruta graveolens* L.	Rutacee			*Trifolium pratense* L.	Fabaceae
*Populus alba* L.	Salicaceae	*Parietaria judaica* L.	Urticaceae			*Anthurium andreanum* Lind.	Araceae
*Iris germanica* L.	Iridoideae	*Cupressus sempervirens* L.	Cupressaceae			*Ficus carica* L.	Moraceae
*Quercus cerris* L.	Fagaceae	*Calycanthus floridus* L.	Calycanthaceae			*Ilex aquifolium* L.	Aquifoliaceae
*Tamarindus indica* L.	Fabaceae	*Picea abies* L.	Pinaceae			*Pyrus communis* L.	Rosaceae
*Salicornia europaea* L.	Amaranthaceae	*Humulus lupulus* L. var. Cascade	Cannabaceae			*Silybum marianum* L.	Asteraceae
*Solanum quitoense* Lam.	Solanaceae	*Humulus lupulus* L.	Cannabaceae			*Portulaca oleracea* L.	Portulacaceae
*Sansevieria trifasciata* Prain.	Asparagaceae	*Hedera helix* L.	Araliaceae			*Capsicum chacoense* Hunz.	Solanaceae
*Diospyros lotus* L.	Ebenaceae	*Cardiospermum halicacabum* L.	Sapindaceae			*Withania somnifera* L.	Solanaceae
*Linaria vulgaris* Mill.	Plantaginaceae	*Curcuma longa* L.	Zingiberaceae			*Rumex acetosa* L.	Polygonaceae
*Taraxacum officinale* F.H. Wigg	Asteraceae	*Foeniculum vulgare* Mill.	Apiaceae			*Punica granatum* L.	Lythraceae
*Wisteria floribunda* (Willd.)	Fabaceae	*Laurus nobilis* L.	Lauraceae			*Nerium oleander* L.	Apocynaceae
*Gossypium herbaceum* L.	Malvaceae	*Magnolia grandiflora* L.	Magnoliaceae			*Iris pallida* Lamm.	Iridaceae
*Mespilus germanica* L.	Rosaceae	*Citrus x Aurantium* L.	Rutaceae			*Hibiscus syriacus* L.	Malvaceae
*Prunus persica* L.	Rosaceae	*Carica papaya* L.	Caricaceae				
*Paulownia tomentosa* Steud.	Paulowniaceae	*Aloe vera* L.	Xanthorrhoeaceae				
*Capsicum annuum* L.	Solanaceae	*Liquidambar styraciflua* L.	Hamamelidaceae				
		*Artemisia vulgaris* L.	Asteraceae				
		*Magnolia liliiflora* (Desr.)	Magnoliaceae				
		*Solanum lycopersicum* L.	Solanaceae				
		*Chrysanthemum indicum* L.	Asteraceae				
		*Tetradenia riparia* (Hochst.) Codd.	Lamiaceae				
		*Crithmum maritimum* L.	Apiaceae				
		*Paullinia cupana* Kunth.	Sapindaceae				
		*Coccoloba uvifera* L.	Polygonaceae				
		*Turnera afrodisiaca* Ward.	Turneraceae				
		*Citrus Limon* L.	Rutaceae				
		*Psidium guajava* L.	Myrtaceae				
		*Lavandula spica* L.	Lamiaceae				
		*Dahlia pinnata* Cav.	Asteraceae				
**Spec**.	**Fam**.	**Spec**.	**Fam**	**Spec**.	**Fam**.	**Spec**.	**Fam**.

Cluster 4 is made up by the isolated nodes, i.e. by all that species which don’t share any VOCs with all the other species.

Cluster 2, besides being the biggest one, is made up by those species corresponding to the highest weighted degree vertices in ${G}_{2}^{P}({V}_{2},\,{E}_{2})$. That species work as highly connected nodes, and they share several VOCs with the other neighboring nodes. They correspond to the biggest yellow nodes in Fig. 4. Here we list the principal ones: *Lavandula spica* L. (Lavander), *Foeniculum vulgare* Mill. (Fennel), *Crithmum maritimum* L. (Samphire), *Liquidambar styraciflua* L. (Sweetgum), *Chrysanthemum indicum* L. (Chrisanth), *Santolina chamaecyparissus* L. (Cotton lavender), *Curcuma longa* L. (Turmeric), *Cupressus sempervirens* L. (Mediterranean cypress), *Ocimum basilicum* L. (Basil), *Citrus x Aurantium* L. (Bitter orange), *Tetradenia riparia* (Hochst.) Codd. (Ginger bush), *Juniperus communis* L. (Juniper), *Artemisia vulgaris* L. (Mugwort), *Citrus x Limon* L. (Lemon), *Stevia rebaudiana* (Stevia), *Eucalyptus globulus* L. (Eucalyptus), *Quercus ilex* L. (Holm oak), *Hedera helix* L. (Ivy).

Other species with as well a huge emission of VOCs are present in cluster 1: *Gossypium herbaceum* L. (Cotton), *Plantago lanceolata* L. (Plantain), and *Inula viscosa* L. (Inula) are the most connected aqua nodes in Fig. 4.

Cluster 3 (violet vertices in Fig. 4) turns out to be the most homogeneous one in terms of families composition, since it groups species belonging mainly to *Brassicaceae* family, characterised by the predominant emission of sulphur compounds.

#### Selected VOCs graph

Communities detection algorithms were applied to the plants graph ${G}_{3}^{P}({V}_{3},\,{E}_{3})$, obtained by taking into account the relation among plants, based on the emission of a reduced subset of VOCs. The same procedure described for ${G}_{2}^{P}({V}_{2},\,{E}_{2})$ graph was followed. The VOCs reduction reflected into a more clear picture of species reciprocal behaviour in terms of emitted protonated masses. Besides the set of 28 isolated nodes, tree big communities were detected. A detailed description of the plants families and species composition of each cluster of ${G}_{3}^{P}({V}_{3},\,{E}_{3})$ graph is provided in Tables 4 and 5, respectively.

**Table 4:** Plants families composition in each community extracted from graph ${G}_{3}^{P}({V}_{3},\,{E}_{3})$ by modularity (BL) algorithm, and the corresponding amount of species belonging to that families for each community.

cluster 1		cluster 2		cluster 3		cluster 4	
Cannabaceae	3	Asteraceae	5	Brassicaceae	4	Solanaceae	3
Polygonaceae	3	Apiaceae	3	Fabaceae	3	Araceae	2
Sapindaceae	3	Lamiaceae	3	Rosaceae	2	Fabaceae	2
Asteraceae	2	Cupressaceae	2	Amaranthaceae	1	Rosaceae	2
Lauraceae	2	Araliaceae	1	Asteraceae	1	Actinidiaceae	1
Magnoliaceae	2	Brassicaceae	1	Betulaceae	1	Apocynaceae	1
Malvaceae	2	Composite	1	Cyperaceae	1	Aquifoliaceae	1
Martyniaceae	2	Fagaceae	1	Ebenaceae	1	Asparagaceae	1
Rosaceae	2	Hamamelidaceae	1	Ericaceae	1	Asteraceae	1
Rutacee	2	Myrtaceae	1	Oleaceae	1	Crassulaceae	1
Solanaceae	2	Pinaceae	1	Plantaginaceae	1	Faboideae	1
Calycanthaceae	1	Poaceae	1	Salicaceae	1	Fagaceae	1
Caricaceae	1	Rosaceae	1	Solanaceae	1	Lythraceae	1
Convolvulaceae	1	Solanaceae	1			Moraceae	1
Ebenaceae	1	Urticaceae	1			Oleaceae	1
Fabaceae	1	Zingiberaceae	1			Paulowniaceae	1
Hydrangeaceae	1					Plantaginaceae	1
Iridaceae	1					Platanaceae	1
Iridoideae	1					Polygonaceae	1
Moraceae	1					Portulacaceae	1
Myrtaceae	1					Rhamnaceae	1
Turneraceae	1					Vitaceae	1
						Xanthorrhoeaceae	1
**Fam**.	# **Spec**.	**Fam**.	# **Spec**.	**Fam**.	# **Spec**.	**Fam**.	# **Spec**.

**Table 5:** Species composition of each cluster found in ${G}_{3}^{P}({V}_{3},\,{E}_{3})$ graph.

cluster 1		cluster 2		cluster 3		cluster 4	
*Cannabis sativa* L.	Cannabaceae	*Ocimum basilicum* L.	Lamiaceae	*Brassica rapa* L.	Brassicaceae	*Zamioculcas zamiifolia* (Lodd.)	Araceae
*Ibicella lutea* L.	Martyniaceae	*Brassica oleracea* L. var botrytis	Brassicaceae	*Cyperus papyrus* L.	Cyperaceae	*Solanum marginatum* L.	Solanaceae
*Proboscidea parviflora* (Woot. Et Standl.)	Martyniaceae	*Stevia rebaudiana*	Asteraceae	*Brassica oleracea* L. var acephala	Brassicaceae	*Vitis vinifera* L.	Vitaceae
*Convolvulus cneorum* L.	Convolvulaceae	*Nicotiana tabacum* L.	Solanaceae	*Plantago lanceolata* L.	Plantaginaceae	*Echeveria elegans* (Rose)	Crassulaceae
*Rheum rhabarbarum* L.	Polygonaceae	*Eucalyptus globulus* L.	Myrtaceae	*Arbutus unedo* L.	Ericaceae	*Rumex acetosella* L.	Polygonaceae
*Ruta graveolens* L.	Rutacee	*Quercus ilex* L.	Fagaceae	*Sonchus oleraceus* L.	Asteraceae	*Acacia dealbata* Link	Fabaceae
*Hydrangea macrophylla* (Lam.)	Hydrangeaceae	*Artemisia dracunculus* L.	Composite	*Corylus avellana* L.	Betulaceae	*Ziziphus jujuba* Mill.	Rhamnaceae
*Persea americana* Mill.	Lauraceae	*Juniperus communis* L.	Cupressaceae	*Osmanthus heterophyllus* (G. Don)	Oleaceae	*Robinia pseudoacacia* L.	Faboideae
*Mimosa pudica* L.	Fabaceae	*Santolina chamaecyparissus* L.	Asteraceae	*Diospyros kaki* L.	Ebenaceae	*Olea europaea* L.	Oleaceae
*Humulus lupulus* L. var. Cascade	Cannabaceae	*Arundo donax* L.	Poaceae	*Populus alba* L.	Salicaceae	*Trifolium pratense* L.	Fabaceae
*Humulus lupulus* L.	Cannabaceae	*Parietaria judaica* L.	Urticaceae	*Tamarindus indica* L.	Fabaceae	Anthurium andreanum Lind.	Araceae
*Rosa chinensis* (Jacq.)	Rosaceae	*Cupressus sempervirens* L.	Cupressaceae	*Salicornia europaea* L.	Amaranthaceae	*Ficus carica* L.	Moraceae
*Cardiospermum halicacabum* L.	Sapindaceae	*Picea abies* L.	Pinaceae	*Eruca sativa* (Mill.)	Brassicaceae	*Ilex aquifolium* L.	Aquifoliaceae
*Silybum marianum* L.	Asteraceae	*Hedera helix* L.	Araliaceae	*Solanum quitoense* Lam.	Solanaceae	*Platanus x Acerifolia* (Willd.)	Platanaceae
*Laurus nobilis* L.	Lauraceae	*Curcuma longa* L.	Zingiberaceae	*Vicia faba* L.	Fabaceae	*Pyrus communis* L.	Rosaceae
*Inula viscosa* L.	Asteraceae	*Foeniculum vulgare* Mill.	Apiaceae	*Wisteria floribunda* (Willd.)	Fabaceae	*Actinidia arguta* (Siebold & Zucc.)	Actinidiaceae
*Magnolia grandiflora* L.	Magnoliaceae	*Cotoneaster horizontalis* Decne.	Rosaceae	*Mespilus germanica* L.	Rosaceae	*Portulaca oleracea* L.	Portulacaceae
*Prunus armeniaca* L.	Rosaceae	*Liquidambar styraciflua* L.	Hamamelidaceae	*Prunus persica* L.	Rosaceae	*Aloe vera* L.	Xanthorrhoeaceae
*Acer campestre* L.	Sapindaceae	*Artemisia vulgaris* L.	Asteraceae	*Lunaria annua* L.	Brassicaceae	*Quercus cerris* L.	Fagaceae
*Calycanthus floridus* L.	Calycanthaceae	*Apium graveolens* L.	Apiaceae			*Fragaria vesca* L.	Rosaceae
*Carica papaya* L.	Caricaceae	*Chrysanthemum indicum* L.	Asteraceae			*Withania somnifera* L.	Solanaceae
*Ficus benjamina* L.	Moraceae	*Tetradenia riparia* (Hochst.) Codd.	Lamiaceae			*Sansevieria trifasciata* Prain.	Asparagaceae
*Iris germanica* L.	Iridoideae	*Crithmum maritimum* L.	Apiaceae			*Linaria vulgaris* Mill.	Plantaginaceae
*Magnolia liliiflora* (Desr.)	Magnoliaceae	*Lavandula spica* L.	Lamiaceae			*Taraxacum officinale* F.H. Wigg	Asteraceae
*Capsicum chacoense* Hunz.	Solanaceae	*Dahlia pinnata* Cav.	Asteraceae			*Punica granatum* L.	Lythraceae
*Solanum lycopersicum* L.	Solanaceae					*Nerium oleander* L.	Apocynaceae
*Paullinia cupana* Kunth.	Sapindaceae					*Paulownia tomentosa* Steud.	Paulowniaceae
*Coccoloba uvifera* L.	Polygonaceae					*Capsicum annuum* L.	Solanaceae
*Turnera afrodisiaca* Ward.	Turneraceae						
*Diospyros lotus* L.	Ebenaceae						
*Citrus x Limon* L.	Rutaceae						
*Citrus x Aurantium* L.	Rutaceae						
*Psidium guajava* L.	Myrtaceae						
*Rumex acetosa* L.	Polygonaceae						
*Gossypium herbaceum* L.	Malvaceae						
*Iris pallida* Lamm.	Iridaceae						
*Hibiscus syriacus* L.	Malvaceae						
**Spec**.	**Fam**.	**Spec**.	**Fam**	**Spec**.	**Fam**.	**Spec**.	**Fam**.

Cluster 4 is made up by the isolated nodes, i.e. all that species which do not emit any VOC.

**Figure 5:**
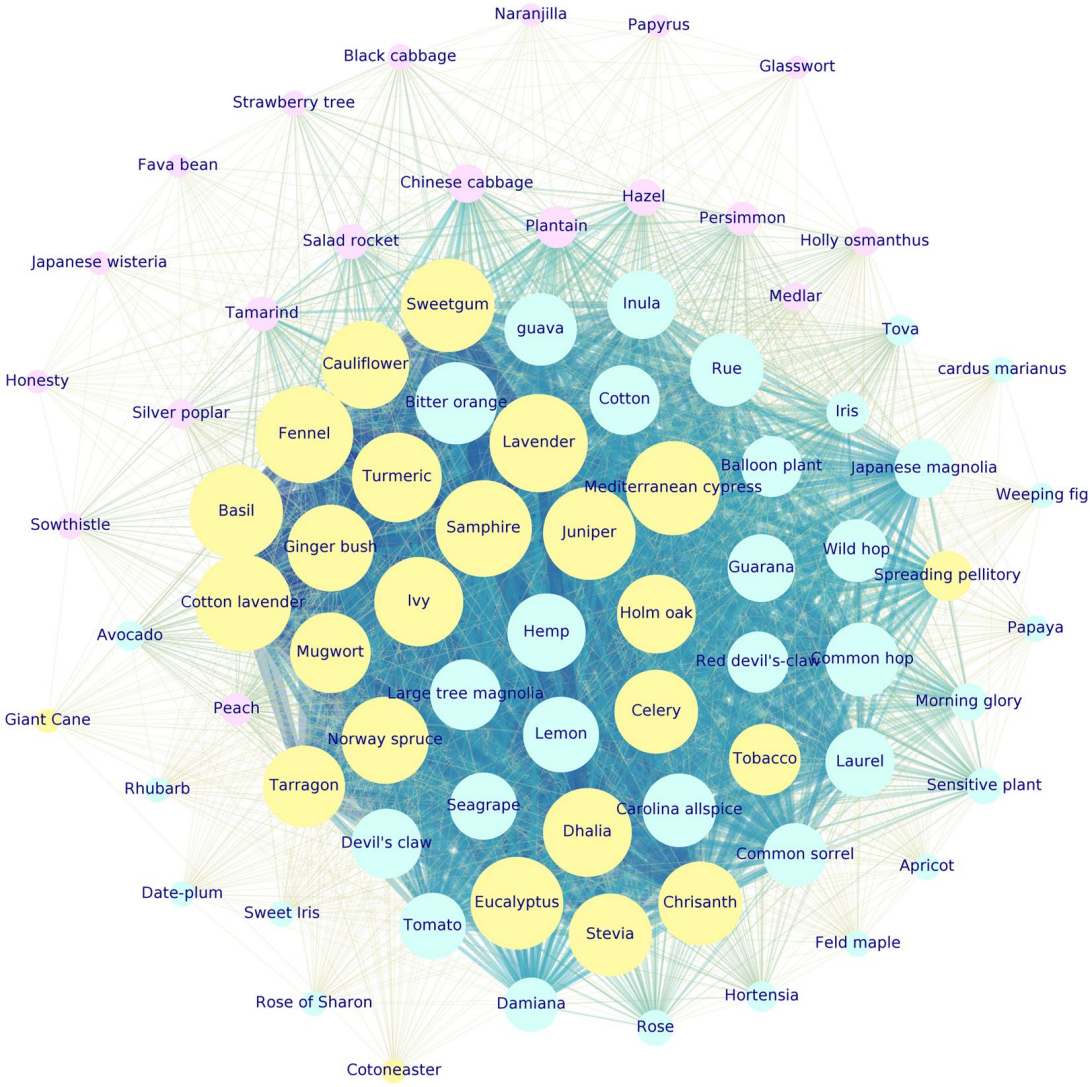
${G}_{3}^{P}({V}_{3},\,{E}_{3})$ selected-VOCs graph. Each color corresponds to one detected community: cluster 1 (aqua), cluster 2 (yellow), cluster 3 (violet). The 28 isolated nodes are not shown (cluster 4). Nodes dimension is proportional to their weighted degree. Edges thickness is proportional to the edge weight. Still *Lavandula spica* L. (Lavender), *Foeniculum vulgare* Mill. (Fennel), *Santolina chamaecyparissus* L. (Cotton lavender), and *Crithmum maritimum* L. (Samphire) are some of the most VOCs emitting species.

Figure 5 shows ${G}_{3}^{P}({V}_{3},\,{E}_{3})$ graph partitioning. The graph’s nodes are coloured according to their community membership. Such as for ${G}_{2}^{P}({V}_{2},\,{E}_{2})$ bipartite projection graph, the biggest nodes correspond to those species which share several VOCs with the other neighboring species. Analogously, edges weights are proportional to the amount of VOCs shared by each couple of adjacent vertices.

Again cluster 2 (yellow nodes, Fig. 5) is made up by the highest-weighted-degree nodes. In other terms the species corresponding to yellow nodes are the most interconnected ones: *Lavandula spica* L. (Lavender), *Foeniculum vulgare* Mill. (Fennel), *Santolina chamaecyparissus* L. (Cotton lavender), *Crithmum maritimum* L. (Samphire), *Cupressus sempervirens* L. (Mediterranean cypress), *Ocimum basilicum* L. (Basil), *Liquidambar styraciflua* L. (Sweetgum), *Eucalyptus globulus* L. (Eucalyptus), *Juniperus communis* L. (Juniper), *Curcuma longa* L. (Turmeric), *Hedera helix* L. (Ivy), *Dahlia pinnata* Cav. (Dhalia), *Brassica oleracea* L. *var botrytis* (Cauliflower), *Picea abies* L. (Norway spruce), *Tetradenia riparia* (*Hochst*.) Codd. (Ginger bush), *Apium graveolens* L. (Celery), *Stevia rebaudiana* (Stevia), *Artemisia dracunculus* L. (Tarragon), *Artemisia vulgaris* L. (Mugwort), *Quercus ilex* L. (Holm oak). That result is fully in agreement with the previous one.

Some highly connected nodes are also present in cluster 1 (aqua nodes, Fig. 5), such as for example: *Citrus x Aurantium* L. (Bitter orange), *Cannabis sativa* L. (Hemp), *Citrus x Limon* L. (Lemon), *Humulus lupulus* L. *var*. *Cascade* (Common hop), *Ruta graveolens* L. (Rue), *Calycanthus floridus* L. (Carolina allspice) and *Psidium guajava* L. (guava).

Cluster 3 is still homogeneously made-up by *Brassicaceae* species (violet vertex in Fig. 5).

Hereafter the four communities are described in term of dominating families and clustering protonated masses.

- cluster 1: it is the biggest community, made up by 37 species (33.9% of the total species amount) grouped into 23 families; dominant families: *Cannabaceae*, *Polygonaceae*, *Sapindaceae*, *Asteraceae*, *Lauraceae*, *Magnoliaceae*, *Malvaceae*, *Martyniaceae*, *Rosaceae*, and *Solanaceae*. This community is characterised by an high heterogeneity in terms of its families composition. The species belonging to that cluster release in particular PM93 (Tp-f, 22 species), PM109 (Tp-f) and PM137 (Tp/STp-f) (26 species), PM95 (STp-f), PM121 (Tp-f), PM123 (Tp/STp-f), PM149 (Tp/STp-f), PM205 (STp) (more than 20 species). The *m*/*z* listed above probably refer to terpenes compounds and almost all of them are found in plant belonging to cluster 2 of the previous ${G}_{2}^{P}({V}_{2},\,{E}_{2})$ graph communities detection analysis. Indeed, the actual cluster 1 shares with the previous cluster 2 more than 51% of plant species (compare Tables 3 and 5), including *Citrus* spp. In this community the species that release sulfur compounds (PM49 and PM63) are also found, such as: *Ruta graveolens* L. (Rue), *Inula viscosa* L. (Inula), *Psidium guajava* L. (Guava), *Gossypium herbaceum* L. (Cotton), and *Citrus x Aurantium* L. (Bitter orange), which together with *Cannabis sativa* L. (Hemp), and *Citrus x Limon* L. (Lemon) are among the most emitting species. Interestingly, species from *Brassicaceae* family, typically rich in sulfur compounds^41^, are not included in this cluster.
- cluster 2: 25 species (22.9% of database total species) grouped in 16 families; prevailing families: *Asteraceae* (5 species), *Apiaceae*, *Lamiaceae*, and *Cupressaceae*. This community is made up by those species which are the most active in terms of VOCs emission, in agreement with the species gathered in cluster 2 of the previous ${G}_{2}^{P}({V}_{2},\,{E}_{2})$ graph analysis; see yellow nodes in Fig. 4 and Table 3. As an example, we just list the most interconnected nodes: *Lavandula spica* L. (Lavender), *Foeniculum vulgare* Mill. (Fennel), *Santolina chamaecyparissus* L. (Cotton lavender, known for its smell), *Crithmum maritimum* L. (Samphire) (found in cluster 2 of the previous analysis). Cauliflower is also found here. Again, an high heterogeneity characterises the families distribution. Accordingly, the species belonging to this cluster release some volatiles already highlighted for the previous cluster 2; in fact, the most released VOC is PM153 (Tp), emitted by 24 species, followed by PM93 (Tp-f), PM95 (STp-f), PM121 (Tp-f), PM123 (Tp/STp-f), PM149 (Tp/STp-f) (released by 23 species), and PM109 (Tp-f), PM119 (Tp-f), PM133 (Tp), PM137 (Tp/STp-f), PM143 (K/Ald), PM151 (Tp/Tp-f), PM205 (STp) (emitted by more than 20 species). Except for the ketone PM143, they are all terpenes compounds.
- cluster 3: 19 species (17.5% of total species) from 13 families only: *Brassicaceae*, *Actinidiaceae*, and *Fabaceae*. All these species emit in particular sulphur compounds PM49 (SC) and PM63 (SC) (13 and 12 species, respectively), while just few of them also release PM93, PM95, and PM153 (Tp-f, STp-f and Tp, respectively). *Brassica rapa* L. (Chinese cabbage) species again distinguishes, being the only one which emits PM143 (K/Ald). This cluster is the most stable and it corresponds to cluster 3 of the previous analysis. It shows an homogenous families composition, since it groups all the *Brassicaceae* species, exception made for the *Brassica oleracea* L. *var botrytis* (Cauliflower) species, in agreement with previous analysis.
- cluster 4: 28 isolated species (25.7% of total species) belonging to 23 different families dominated by *Solanaceae*, *Araceae*, *Fabaceae*, *Rosaceae*. As for the previous analysis on graph ${G}_{2}^{P}({V}_{2},\,{E}_{2})$ the isolated nodes correspond to species which do not emit any VOC.

#### VOCs graph, ${G}_{3}^{PM}$

**Figure 6:**
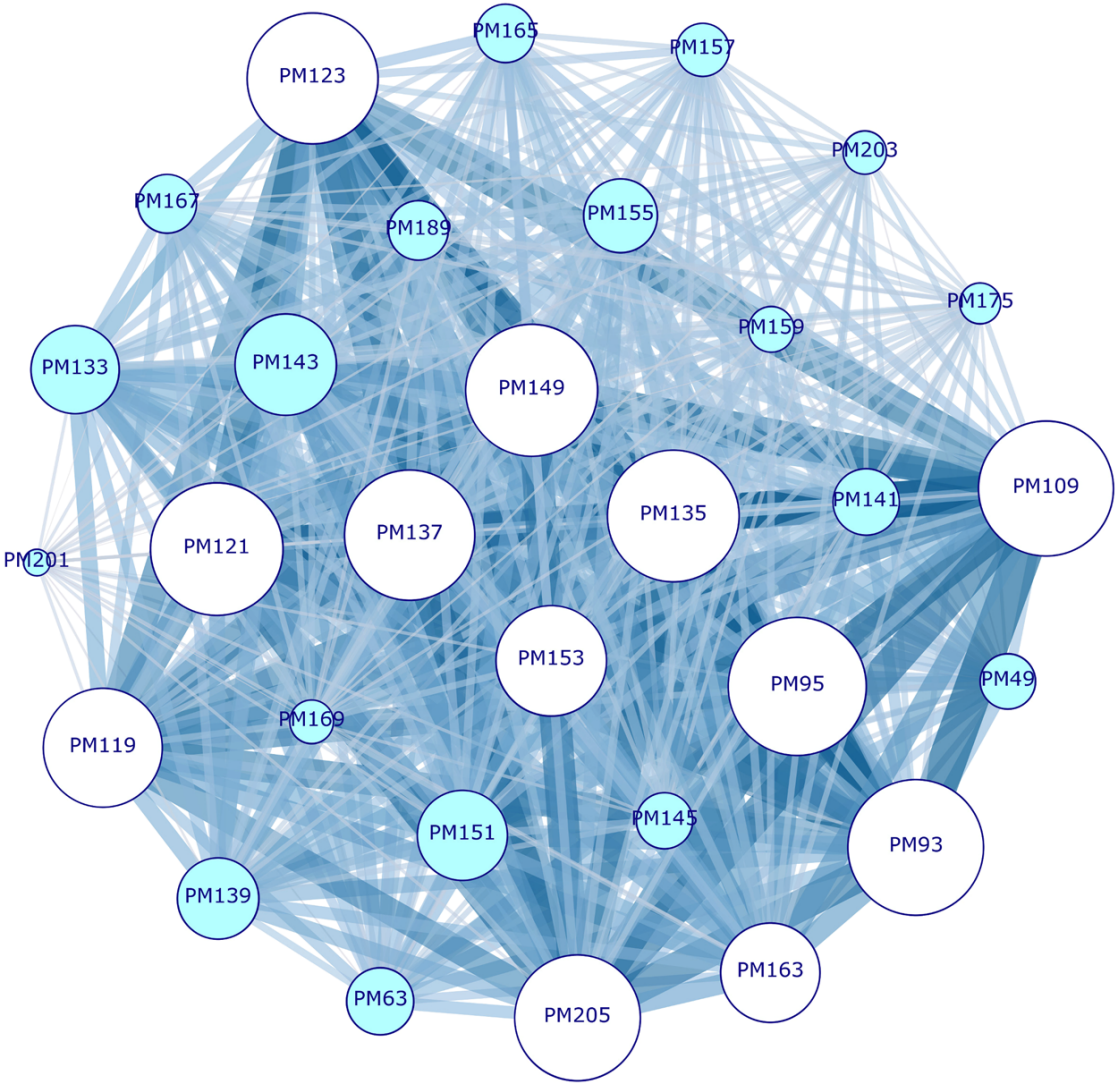
${G}_{3}^{PM}({V}_{3b},\,{E}_{3b})$ features graph. Each vertex correspond to one VOC. Edges thickness is proportional to the amount of shared species. Nodes dimensions are proportional to their weighted degree. The most interconnected VOCs are evident (lighter color): PM93 (Tp-f), PM95 (STp-f), PM109 (Tp-f), PM121 (Tp-f), PM123 (Tp/STp-f), PM135 (Tp/STp-f), PM137 (Tp/STp-f), PM149 (Tp/STp-f), PM205 (STp). The protonated mass PM201 (Tp), on the contrary, is the less interconnected node, thus turning out to be the less commonly shared emitted VOC.

The second bipartite projection of graph *G*_3_(*V*, *E*), i.e. the VOCs-to-VOCs graph of selected protonated masses ${G}_{3}^{PM}({V}_{3b},\,{E}_{3b})$, is shown in Fig. 6. The graph is made up by *V*_3*b*_ = 30 vertices (each corresponding to one protonated mass), and *E*_3*b*_ = 435 edges. Usually a bipartite graph is based on the representation of different individuals according to the common properties they share. Here the emitted VOCs are the analogous of features, since the most two plants emit the same volatiles the most they are similar. More in details, the VOCs are the vertices of the network ${G}_{3}^{PM}({V}_{3b},\,{E}_{3b})$: two VOCs are connected if there is at least one plant emitting both of them. The weight of the link connecting the two VOCs is proportional to the number of plants emitting both of them. We show only the results coming from the second bipartite projection of graph *G*_3_(*V*, *E*), since we obtained similar results for *G*_2_(*V*, *E*). Graph *G*_1_(*V*, *E*) is not considered since from the previous analyses it turned out to be less suitable to describe our experimental data as a network.

Colors here help the reader to distinguish between the most and less interconnected VOCs. Such as for the species-based bipartite projection graph, some protonated masses are highly connected with their neighborhoods. The highest value of weighted degree is recorded for PM95 (*s*_*max*_ = 679), followed by PM93, PM109, PM121, PM149, PM135, PM123, PM137, PM205 (light blue vertices in Fig. 6). All that VOCs are shared by a large number of species, and they are terpenes compounds; accordingly, they are the responsible for the species grouping in the first two communities of graph ${G}_{3}^{P}({V}_{3},\,{E}_{3})$ (aqua and yellow clusters in Fig. 5), made up by species rich in such types of compounds. Indeed, terpenes are the largest and assorted group of plant natural products, including hemiterpenes (C_5_), monoterpenes (C_10_), sesquiterpene (C_15_), homoterpenes (C_11_ and C_16_), some diterpene (C_20_) and triterpene (C_30_), that are easily released into the atmosphere. The highest amount of species shared between two VOCs is observed between all the following couples of VOCs: PM93–PM95 and PM93–PM109 (respectively 48 and 46 maximum numbers of species), PM95–PM109, PM109–PM137, PM93–PM121, PM95–PM121, PM95–PM135, PM95–PM137, PM95–PM149, PM109–PM121, PM93–PM123. Their corresponding links are the thickest ones (highest link weights) in Fig. 6. In most cases, plants share two compounds belonging to the same chemical class; for example, PM95–PM109 is a couple of sesquiterpenes and/or sesquiterpenes fragments, while PM93–PM123 are terpenes and/or terpenes fragments. It’s worth noting that sesquiterpenes have a distinct biochemical pathway from that of other hemiterpenes^44^, thus it is more expectable that a plant species emits, simultaneously, two or more VOCs of the same class instead of the combination of VOCs of different classes. However, terpenes biosynthesis is very complex^45^ and uses many separated pathways, and cases of plants producing isoprene (terpenes building unit) but not other monoterpenes (and viceversa) have been frequently reported^46^.

On the contrary, the two sulphur compounds PM49 and PM63, which considerably determine the assembling of the violet cluster in Fig. 5, are small dimension nodes, since the species they share are homogeneous in term of family composition, but they are few. Among volatile organic sulfur compounds, dimethylsulfide (DMS, PM63) and methanethiol (MT, PM49) are two of the most frequent products of plant metabolism. Their biosynthetic pathways share the role of a common lyase enzyme (dimethylsulfoniopropionate, DMSP) that is not widely distributed in terrestrial plants^47^.

Finally, PM201 (Tp), PM169 (aldehydes, Ald, a product of monoterpene oxidation), and PM159 (acids/esters, Ac/Es) are some of the less interconnected VOCs.

## Conclusions

Volatile organic compounds (VOCs), that represent a crucial component of a plant’s phenotype^3^, have been analysed by bipartite networks methodology in order to classify plants species. In particular, several quantitative measures coming from Complex Network Theory^23–25^ have been applied to uncover eventual similarities between the species in term of their VOCs emissions. To assure the reliability and robustness of the results, different classical and advanced community detection algorithms have been applied, and only the comparable results were retained. Moreover data have been pre-processed by means of both descriptive and quantitative statistical methods, to better focus on data behaviour. VOCs time series, obtained by recording the emissions content for each available species, suggest the presence of spike-like pulses (corresponding to few species), exceeding from a quite flat background signal. Each VOC turns out to be emitted by few species in a very large quantity with respect to all the other species emissions of the same protonated mass.

After a preliminary test performed on the whole dataset, some VOCs have been excluded. In fact, some volatiles, especially C6 compounds and acetaldehyde, can occur in response to external stress, including wounding; this should be taken into account when using these compounds for communities detection analysis. Using a reduced dataset, community detection suggested the presence of 4 clusters. Two communities are made up by highly VOCs-emitting species. We recall here the most interconnected nodes: *Lavandula spica* L. (Lavender), *Foeniculum vulgare* Mill. (Fennel), *Santolina chamaecyparissus* L. (Cotton lavender), *Crithmum maritimum* L. (Samphire), *Cupressus sempervirens* L. (Mediterranean cypress), *Ocimum basilicum* L. (Basil) (for cluster 1); *Citrus x Aurantium* L. (Bitter orange), *Cannabis sativa* L. (Hemp), *Citrus x Limon* L. (Lemon), *Humulus lupulus* L. *var*. *Cascade* (Common hop), *Ruta graveolens* L. (Rue), *Calycanthus floridus* L. (Carolina allspice) and *Psidium guajava* L. (guava) (for cluster 2). A third community clearly groups species belonging to *Brassicaceae* family, turning out to be quite homogeneous in terms of clusters families composition. Finally, a fourth community highlights all those species which, by network construction, are not sharing any VOCs emission with the other species. See previous Section “Community detection analysis” for more details.

The second bipartite projection confirmed terpenes compounds and sulphur compounds to be the two chemical classes most responsible for species classification. Indeed, the chemistry of volatiles has been shown to be species-specific^44^; for example, species characterised by terpenes and nitrogen-containing compounds as floral volatiles are different from species releasing sulphur-containing volatiles^48^. Moreover, terpenes compounds emitted by plant species (the so-called “terpenome”^49^), are the major constituents of plants essential oils^50^, and can be used to distinguish different species; in this study, although the exact chemical definition of the compounds involved is beyond the purpose, community detection highlighted two well defined groups (clusters 1 and 2) of species that emit different terpenes compounds. It emerges quite clearly that many VOCs are common to phylogenetically distant plant species, suggesting that the metabolic pathways of their production have been preserved during times. Further investigation on the emission of volatiles in species representative of plant evolution will give insight on VOCs involvement with specific functions that appear at specific moments of the evolution, thus allowing to obtain more information on the biological sense of plants clustering.

In conclusion, complex network analysis allows to measure and describe hidden plants relationships probably related to the way they react to their environment. That result strengthens previous findings obtained by applying Complex Network Theory to plants morphological features^33^. A better understanding of the plant relationships would take further advantages from the correlation between VOCs emitted by different plant organs. Such approach could be then extended to different fields in botanic framework, such as plant ecology, psychophysiology and plant communication.

## Methods

### Data

PTR-ToF-MS has been used in this study as the detector for the organic compounds emitted by leaf samples. A full description of this tool, with its advantages and disadvantages, can be found elsewhere^51–53^. The compounds emitted by different leaves were transported from the air stream where collided with H_3_O^+^ reagent ion inside the drift tube. The analysis was carried out as follows: each leaf samples was placed into 3/4 L glass jar (Bormioli, Italy) provided of glass stopper fitted with two Teflon tubes connected respectively to the PTR-ToF-MS (8000, Ionicon Analitic GmbH, Innsbruck, Austria) and the zero air generator (Peak Scientific instruments, USA). An overview of the plants used is shown in Tables 3 and 5, for a total of 109 species belonging to 56 plant families. Plant database was chosen as wide as possible in order to assure the statistical robustness of the results. Particular attention was put to include plant species representative of different climates (Mediterranean and Tropical), growth (woody and herbaceous plants), and domestication (domesticated and wild). Most of the ornamental plants were collected in an open field belonging to one of the biggest European nursery close to our laboratory (Innocenti and Mangoni Piante, Pistoia, Italy); tropical plants came from the greenhouse collection of a local association (Marco Billi, Shangri-La Association, Florence, Italy); the remaining ones, including cultivated trees and vegetables, were available at the germplasm collection of DISPAA (University of Florence, Italy). In all locations, plants were grown according to their specific needs in terms of temperature, light, nutrients, water and pest management. Adult plants were moved from the greenhouses or open fields to a growth chamber adjacent the PTR-ToF-MS laboratory (at least 3 days before VOCs measurements). Here, temperature and light (Sylvania Gro-Lux^®^ tube) were kept at 24 ± 1 °C and 12/12 hours day/night, respectively, allowing to obtain leaf samples from non-stressed plants grown under stable conditions. In order to standardise the procedure, representative mature and healthy leaves were harvested from three plants for each species (15, 9 and 3 leaves respectively for plant species with small and light leaves, medium leaves, big and thick leaves). Leaves were cut in four pieces with a sterile razor perfectly shaved, in order to avoid excessive damages of the tissues. Cut samples of each plant species were divided in three replicates, weighted separately and analysed. PTR-ToF-MS results were then referred to the weight unity (1 g). Before each leaf sample analysis, the glass jar was exposed to 1 minute of purified air flux (100 sccm) to remove all the VOCs accumulated in the head space during the time between sample preparation; then, a blank air sample was taken and subsequently used for background correction. All measurements were conducted in an air-conditioned room, with temperature and humidity respectively set at 20 ± 3 °C and 65%^54^, and using the same PTR-ToF-MS instrumental parameters: drift pressure = 2.30 mbar, drift temperature = 60 °C and inlet temperature = 40 °C, drift voltage = 600 V, extraction voltage at the end of the tube (Udx) 35 V, which resulted in E/N ratio of 140 Td (1 Td = 10–17 V cm^−2^). This setup allowed a good balance between excessive water cluster formation and product ion fragmentation^55^. Moreover, the inlet flux was set to 100 sscm. The internal calibration of ToF spectra was based on m/z = 29.997 (NO^+^), m/z = 59.049 (C_3_H_7_O^+^) and m/z = 137.132 (C_10_ ${{\rm{H}}}_{17}^{+}$) and was performed off-line after dead time correction; for peak quantification, the resulting data were corrected according to the duty cycle. Data were recorded with the software TOF-DAQ (Tofwerk AG, Switzerland), the sampling time for each channel of TOF acquisition was 0.1 ns, acquiring 1 spectrum per second, for a mass spectrum range between m/z 20 and m/z 220. The raw data were normalised to the primary ion signal from counts per seconds (cps) to normalised counts per second (*n*cps) as described by *Herbig et al*.^56^. Data were filtered following the procedure used by *Taiti et al*.^57^ and used for statistical analysis. In this manner, a dataset comprised of mean mass spectra for each sample analysed was compiled. Finally, the tentative identifications of peaks was performed on the basis of an high mass resolution and rapid identification of compounds with a high level of confidence^58^. Further characterization of VOCs belonging to certain chemical classes such as terpenes, which are prone to fragmentation, was attempted using literature data on fragmentation of standards during PTR-ToF-MS analysis^59–61^. Similar approach was performed for the other identified compound, e.g. following *Papurello et al*.^62^ and *Liu et al*.^63^ for sulfur compounds, *Loreto et al*.^36^, *Brilli et al*.^37^, *Degen et al*.^38^, and *Wu et al*.^39^ for wounding-related VOCs, and *Schwartz et al*.^64^ and *Soukoulis et al*.^65^ for aldehydes, ketones and alcohols.

### Descriptive statistics: boxplots

Boxplots are an intuitive graphical non-parametric method particularly suitable to visualise the distribution of continuous univariate data, firstly proposed by *Tukey* ^34^. None a-priory assumption is made on the underlying statistical distribution. Boxplots show information about data location and spread, by starting from the estimation of the second quartile (or median, *Q*_2_) and of the interquartile range (*IQR*), where *IQR* = *Q*_3_ − *Q*_1_, and *Q*_3_ and *Q*_1_ are the third and first quartiles, respectively. Boxplots are also known as box-and-whisker plots. The rectangular box is related to the data quartiles, and, more in details, the left and right sides of the rectangle correspond respectively to *Q*_1_ and *Q*_3_. The whiskers are lines extending from the box till lower and upper first outliers. It follows that the boxplot width visually shows the sample *IQR*, the vertical band drawn inside the box represents the median, and as a whole the box is a measure of the data dispersion and skewness. On the contrary, there is no common definition for the end of the boxplots whiskers. In the present work we adopt the following formalism: outliers are defined as those data points lying outside the range (*Q*_1_ − 1.5 × *IQR*; *Q*_3_ + 1.5 × *IQR*); extreme events are defined as those data points exceeding the range (*Q*_1_ − 3 × *IQR*; *Q*_3_ + 3 × *IQR*). Several graphical solutions for boxplots are present nowadays, and generalised versions allow to apply them to skewed distributions, also, by assuring a robust measure of the skewness in the determination of the whiskers^35^. We recall here that the quartiles are also called quantiles of order 1/4, 1/2, 3/4, or ${Q}_{\tfrac{1}{4}}$, ${Q}_{\tfrac{1}{2}}$, and ${Q}_{\tfrac{3}{4}}$, respectively. That second formalism will be used along the paper.

### Building the graph: projection in the space of plants/VOCs

Data are represented as an undirected bipartite graph *G*(*N*, *E*), where every plant species *p* is connected to its features, i.e. in that case the VOCs it emits. No connection is present between the two set of nodes, i.e. the plant species and the recorded VOCs. Usually, a bipartite graph can also be described by a binary matrix *A*(*p*, *f*) whose element *a*_*ij*_ is 1 just if plant *p* shows the feature *f*. The most immediate way to measure correlation between species is counting how many VOCs the plants species share in term of significant emissions, and similarly how many plants emit the same VOCs. We refer to the Basic Network Analysis subsection for a proper description of the methodology. In formulas, this corresponds to consider the matrix of species *P*(*p*, *p*) = *AA* ^*T*^ and the matrix of volatile organic compounds, *F*(*f*, *f*) = *A* ^*T*^ *A*, i.e. the two bipartite projections of *G*(*N*, *E*). In the present work, we focused on the graph having as nodes the different plants, i.e. on the *Plants graph G* ^*P*^(*N*, *E*) whose edges weights are proportional to the number of commonly emitted VOCs between plants. Second, in order to catch the predominant similarities in terms of volatile organic compounds emissions, we analysed the second bipartite projection, i.e. the *Features graph*, *G* ^*F*^(*N*, *E*), whose nodes represent the emitted VOCs. In that case edges weights were proportional to the number of plants sharing the same emitted compound.

### Basic network analysis

As regards network analysis, we computed some global and local basic metrics described hereafter.

- *Graph density* (*D*) is defined as the ratio between the numbers of existing edges and the possible number of edges. Given a *N*-order network, graph density is computed as $D=\tfrac{2E}{N(N-1)}$. Strictly connected to *D*, is the graph average degree $\overline{k}=\frac{1}{V}\,{\sum }_{i=1}^{V}\,{k}_{i}=\tfrac{2E}{V}$, where *k*_*i*_ is the degree of each vertex in *V*, i.e. the number of edges incident to it.
- *Network clustering coefficient* (*c*) is the overall measure of clustering in a undirected graph in terms of probability that the adjacent vertices of a vertex are connected. More intuitively, global clustering coefficient is simply the ratio of the triangles and the connected triples in the graph. The corresponding local metric is the *local clustering coefficient*, which is the tendency among two vertices to be connected if they share a mutual neighbour. In this analysis we used a local vertex-level quantity^20^ defined in Eq. (1):
  1 $${c}_{i}^{w}=\frac{1}{{s}_{i}({k}_{i}-\mathrm{1)}}\,\sum _{jh}\,\frac{({w}_{ij}+{w}_{ih})}{2}{a}_{ij}{a}_{ih}{a}_{jh},$$
  The normalization factor $\tfrac{1}{{s}_{i}({k}_{i}-1)}$ accounts for the weight of each edge times the maximum possible number of triplets in which it may participate, and it ensures that $0\le {c}_{i}^{w}\le 1$. That metric combines the topological information with the weight distribution of the network, and it is a measure of the local cohesiveness, grounding on the importance of the clustered structure evaluated on the basis of the amount of interaction intensity actually found on the local triplets^20^.
- *Network strength* (*s*) is obtained by summing up the edge weights of the adjacent edges for each vertex^20^. That metric is a more significant measure of the network properties in terms of the actual weights, and is obtained by extending the definition of *vertex degree* ${k}_{i}={\sum }_{j}\,{a}_{ij}$, with *a*_*ij*_ elements of the network adjacent matrix **A**. In formulas, ${s}_{i}={\sum }_{j=1}^{N}\,{a}_{ij}{w}_{if}$.

### Grouping plants from graph: communities detection analysis

Communities detection aims essentially at determine a finite set of categories (clusters or communities) able to describe a data set, according to similarities among its objects^66^. More in general, hierarchy is a central organising principle of complex networks, able to offer insight into many complex network phenomena^67^. In the present work we adopted the following methods belonging to complex networks framework:

- **Fast greedy** (**FG**) hierarchical agglomeration algorithm^68^ is a faster version of the previous greedy optimisation of modularity^22^. FG gives identical results in terms of found communities. However, by exploiting some shortcuts in the optimisation problem and using more sophisticated data structures, it runs far more quickly, in time *O*(*md* log *n*), where *d* is the depth of the “dendrogram” describing the network community structure.
- **Walktrap community finding algorithm** (**WT**) finds densely connected subgraphs from a undirected locally dense graph *via* random walks. The basic idea is that short random walks tend to stay in the same community^69^. Starting from this point, *WT* is a measure of similarities between vertices based on random walks, which captures well the community structure in a network, working at various scales. Computation is efficient and the method can be used in an agglomerative algorithm to compute efficiently the community structure of a network.
- **Louvain or Blondel method** (**BL**)^70^ to uncover modular communities in large networks requiring a coarse-grained description. *Louvain* method (*BL*) is an heuristic approach based on the optimisation of the modularity parameter (*Q*) to infer hierarchical organization. Modularity (Eq. (2)) measures the strength of a network division into modules^22, 71^, as it follows:
  2 $$Q=\frac{1}{2m}\sum _{vw}\,[{A}_{vw}-\frac{{k}_{v}{k}_{w}}{(2m)}]\,\delta \,({c}_{v},{c}_{w})=\sum _{i=1}^{c}\,({e}_{ii}-{a}_{i}^{2}),$$
  where, *e*_*ii*_ is the fraction of edges which connect vertices both lying in the same community *i*, and *a*_*i*_ is the fraction of ends of edges that connect vertices in community *i*, in formulas: ${e}_{ii}=\tfrac{1}{2m}\,{\sum }_{vw}\,[{A}_{vw}\delta \,({c}_{v},\,{c}_{w})]$, and ${a}_{i}=\tfrac{{k}_{i}}{2m}={\sum }_{i}\,{e}_{ij}$; **A** is the adjacent matrix for the network; *c* the number of communities; ${k}_{i}={\sum }_{w}\,{A}_{vw}$ the degree of the vertex-*i*, *n* and $m=\tfrac{1}{2}\,{\sum }_{vw}\,{A}_{vw}$ the number of graph vertices and edges, respectively. *Delta* function, *δ*(*i*, *j*), is 1 if *i* = *j*, and 0 otherwise.
- **Label propagation** (**LP**) community detection method is a fast, nearly linear time algorithm for detecting community structure in networks^21^. Vertices are initialised with a unique label and, at every step, each node adopts the label that most of its neighbours currently have, that is by a process similar to an ‘updating by majority voting’ in the neighbourhood of the vertex. Moreover, *LP* uses the network structure alone to run, without requiring neither optimisation of a predefined objective function nor *a*-*priori* information about the communities, thus overcoming the usual big limitation of having communities which are implicitly defined by the specific algorithm adopted, without an explicit definition. In this iterative process densely connected groups of nodes form a consensus on a unique label to form communities.

Besides the complex networks communities detection methodologies, a classic cluster analysis^72, 73^ based on dimensionality reduction methods was also performed to assure the results robustness and reliability, by rejecting those solutions not independent from the statistical methodology applied.
